# Endothelial gateways for brain lipid uptake and metabolism

**DOI:** 10.1172/JCI198352

**Published:** 2025-10-01

**Authors:** A. Dushani Ranasinghe, Timothy Hla

**Affiliations:** Vascular Biology Program, Boston Children’s Hospital, Department of Surgery, Harvard Medical School, Boston, Massachusetts, USA.

## Abstract

Lipids, which constitute half of the brain’s solid matter, are essential for forming specialized membranes of neural cells, providing energy sources, and facilitating cell-to-cell communication. Although the blood-brain barrier restricts lipid movement between peripheral circulation and the brain, multiple mechanisms supply the building blocks necessary to synthesize the diverse lipid species present in the central nervous system (CNS). In this issue of the JCI, Song et al. characterize specialized microvascular niches that metabolize circulating triglyceride-rich lipoproteins (TRLs) to deliver fatty acids into the brain. They located GPIHBP1, an essential chaperone for lipoprotein lipase (LPL) in the fenestrated endothelial cells of the choroid plexus (ChP) and circumventricular organs (CVOs), demonstrating lipolytic processing of peripheral TRLs and brain uptake of fatty acids. This advance implicates the GPIHBP1/LPL lipid metabolic hub in supporting the roles of the ChP and CVO in cerebrospinal fluid composition, immunity, satiety, thirst, and metabolic homeostasis.

Despite comprising only about 2% of body weight, the brain receives 15%–20% of total cardiac output (1), which is necessary to fuel the brain’s high demand for energy. Glucose is the brain’s primary energy source, but lipids are also used to generate ATP for energy-hungry neural cells (2–4). Moreover, tens of thousands of diverse lipid species in the brain carry out myriad functions, including the formation of complex cell and organelle membranes, provision of energy source via ß-oxidation, and communication between cells via lipid signaling pathways (5). For instance, the fatty acid palmitate (16:0) is used as a building block for sphingolipids (e.g., sphingomyelin and glycosphingolipids), which are highly abundant in the brain (6). Unsaturated fatty acids such as oleic acid (18:1), linoleic acid (18:2; n-6), and α linoleic acid (18:3; n-3) are important for the synthesis of phospholipids, which are critical for cell and organelle membrane homeostasis (7).

The spatial distribution and concentration of various lipid molecules within the CNS are precisely controlled, in part by the blood-brain barrier (BBB), a network of endothelial cells connected by tight junctions that line the CNS vasculature and regulate the transfer of molecules and ions from the bloodstream into the brain (8). The BBB restricts the passage of large lipoprotein particles, including triglyceride-rich lipoproteins (TRLs) (9). Therefore, most of the lipids of the brain are synthesized within the CNS, and only some are imported from the periphery (9, 10).

The BBB’s selective transport restrictions pose a challenge in the adequate supply of lipid building blocks. As we will discuss below, it has been widely assumed that TRL-derived lipids are not taken up by the CNS, as CNS endothelial cells do not express glycosylphosphatidylinositol-anchored high-density lipoprotein binding protein-1 (GPIHBP1), an essential chaperone to transfer the lipoprotein lipase (LPL) enzyme from the parenchyma to the apical glycocalyx of the capillary network (11). However, Song et al. show that spatial niches of specialized endothelium in the brain mediate TRL metabolism and lipid uptake, providing metabolic flexibility and access to peripheral lipids otherwise restricted by the BBB (Figure 1) (12).

## Endothelial metabolism of TRLs

In peripheral tissues, lipid uptake requires TRLs in circulating lipoprotein particles (chylomicrons and VLDL) to be processed by the LPL enzyme within the intravascular lumen (13, 14). LPL secreted by adipocytes, myocytes, and other parenchymal cells is shuttled to the glycocalyx of the capillary endothelial cells by the chaperone protein GPIHBP1 (11, 15, 16). GPIHBP1-bound LPL then hydrolyses triglycerides within the core of the circulating TRLs into free fatty acids and glycerol (17–19). This process enables the delivery of fatty acids to the underlying tissues. TRL margination along capillary walls facilitates intimate contact with LPL, optimizing lipid hydrolysis. This process is highly efficient in tissues that depend on ß-oxidation of fatty acids for energy production, such as the heart and the muscle (14). In the absence of endothelial GPIHBP1, LPL remains in interstitial spaces, and unprocessed TRLs accumulate in blood circulation, unable to be delivered to tissues (11).

In the CNS, LPL is made by various neural cells and secreted into the interstitial space of the CNS parenchyma. However, GPIHBP1 is not expressed by the endothelial cells of the BBB, and it has been assumed that TRL metabolism does not supply fatty acids into the brain. Instead, the MFSD2A transporter expressed on BBB endothelium mediates lysophosphatidylcholine uptake. This has been assumed to be the source of fatty acids, including polyunsaturated n-3 and n-6 fatty acids into the brain (20). However, bulk transport of fatty acids into the brain has been enigmatic.

## Importance of TRL margination in the Choroid Plexus

Recent advances have highlighted the choroid plexus (ChP) as a dynamic interface that influences neuronal development, brain metabolism, and immune surveillance, in addition to its well-known function of cerebrospinal fluid (CSF) production (21). The ChP’s ability to sense circulating factors, modulate the brain’s internal environment, secrete growth and signaling factors, and serve as a gateway for peripheral signals and nutrients underscores its essential roles in brain health. Moreover, the ChP performs clearance and filtering roles for the CNS that are analogous to the kidney and liver in the periphery, removing waste, regulating molecular exchange, and maintaining homeostasis within the brain’s internal environment (22).

**Figure 1:**
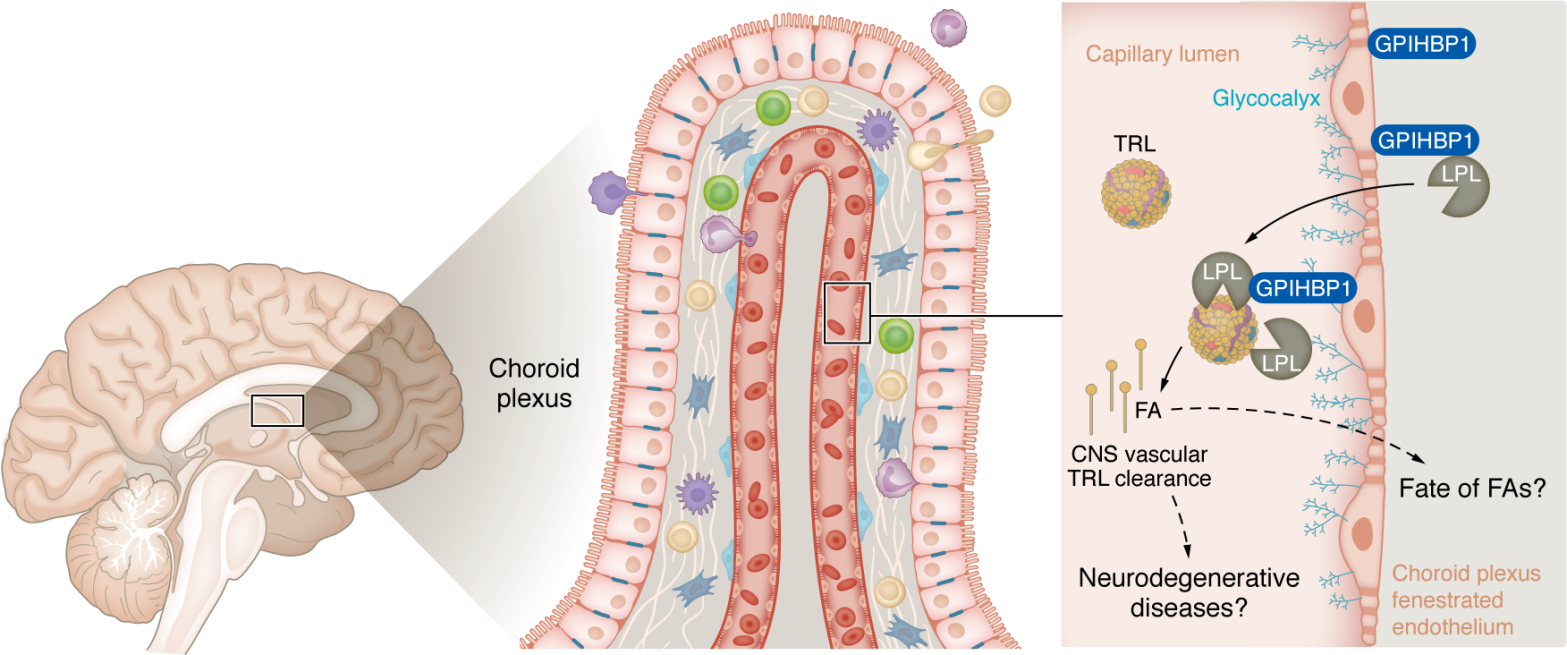
Schematic illustration of fatty acid uptake at the choroid plexus blood-brain barrier. The choroid plexus (ChP) forms a network lining capillaries within the brain’s ventricles. The endothelium of the ChP is characterized by specialized pores called fenestrae that selectively exchange substances between the blood and tissue. Work by Song et al. revealed that circulating triglyceride-rich lipoproteins (TRLs) in the blood interact with endothelial lipoprotein lipase (LPL) on the luminal surface glycocalyx, a process that requires the chaperone GPIHBP1. LPL hydrolyzes TRLs, releasing fatty acids (FAs) that cross the fenestrated ChP endothelium into the tissue. This process highlights a specialized CNS vascular mechanism for lipid uptake and signaling at the ChP. The fate of released FAs, their role in disease (such as Alzheimer’s disease), and mechanisms underlying TRL clearance are valid and important questions to be answered in the future.

The ChP is composed of a single layer of specialized cuboidal epithelial cells (the choroid epithelium) and a central stroma containing loose connective tissue and a dense core of capillaries (Figure 1). These capillaries are fenestrated, meaning that specialized pores called fenestrae in their plasma membrane allow selective exchange of substances between the blood and the surrounding tissue. The fenestrated ECs are surrounded by a basement membrane, facilitating the movement of water, ions, and selected molecules, which are essential for CSF production. The stroma also contains pericytes, connective tissue elements, and various immune and support cells, providing the structural and functional foundation for the ChP (21).

Fenestrated capillaries are also present in the subfornical organ and median eminence, two regions in the CNS with greater access to the peripheral circulation important for sensing systemic nutritional, hormonal, and stress signals (23). Collectively referred to as circumventricular organs (CVOs), such brain structures are essential for physiological homeostasis, including regulation of fluid electrolyte balance, immunity, satiety, and thirst, among others (24, 25).

In the present work, Song et al. showed that the human and mouse fenestrated endothelium of the ChP and CVOs expresses GPIHBP1, which enables the margination and enzymatic processing of TRLs by LPL in CNS vasculature. Consequently, fenestrated endothelium in these regions facilitates local lipid uptake and signaling that was previously considered absent in the CNS vessels with the BBB. The authors used the Secondary Ion Mass Spectrometry (SIMS) technique to demonstrate lipid transport by imaging the distribution of lipids within the ChP capillaries. Their work demonstrated that intravascular lipolytic processing of TRLs occurs within the ChP and CVO microvasculature, enabled by endothelial GPIHBP1, which transports fibroblast-derived LPL to the luminal glycocalyx of the fenestrated capillaries (11, 12, 15, 16).

The mechanism described by Song and colleagues provides the ChP and CVOs with an abundant supply of fatty acids, which may serve as alternative energy substrates, signaling molecules that modulate various brain functions (i.e., eicosanoids), and building blocks for complex lipids (i.e., sphingolipids) (6, 26, 27). Whether LPL-derived fatty acids signal acutely via fatty acid sensors (e.g., GPCRs and nuclear receptors) in the brain is not known as yet but will be important to determine in the future. Furthermore, the kinetics of lipid metabolic flux, whether such lipids function locally in ChP and CVOs, or if they impact distant brain regions, is also not known and will be of interest in future investigations.

## Conclusion and future direction

In summary, the work by Song et al. emphasizes the importance of studying lipid metabolism in specialized CNS vascular niches and expands our understanding of how the brain interfaces with peripheral lipid metabolic and transport systems. It reinforces the ChP’s emerging identity as an active regulatory and metabolic organ beyond CSF production, providing new avenues to explore lipid-related brain physiology and pathology. Investigating these mechanisms may prove especially valuable in neurodegenerative diseases such as Alzheimer’s disease, where disturbances in lipid metabolism and impaired clearance of toxic lipid or protein species contribute to disease progression.

## Version 1 10/01/2025

Electronic publication
